# Role of cardiac mast cells in cardiovascular diseases: a review

**DOI:** 10.7717/peerj.21320

**Published:** 2026-05-22

**Authors:** Li-min Wang, Ling-feng Ye, Jing Wei, Zhi-ying Jiang, Hong-xiang Lu

**Affiliations:** 1 Department of Clinical Laboratory, Jiangning Hospital Affiliated to Nanjing Medical University, Nanjing, China; 2 Department of Clinical Laboratory, Nanjing First Hospital, Nanjing, China

**Keywords:** Cardiac mast cells, Cardiac injury, Cardiovascular disease, Cardiac fibrosis

## Abstract

Mast cells are multifunctional immune cells that play complex roles in maintaining tissue homeostasis and disease pathogenesis. They are also involved in inflammation, lymphangiogenesis, and tissue remodeling by releasing several inflammatory mediators. Recent preclinical studies highlighting the heterogeneity of cardiac mast cells (CMCs) have intensified the focus on elucidating their functional diversity in both tissue homeostasis and disease pathogenesis. Notably, CMCs can exhibit distinct or even opposite functions to adapt to the dynamic tissue environment. Although the precise mechanisms remains unclear, evidence from animal studies suggests that mast cells participate in human diseases in ways similar to macrophages and other inflammatory cells, either directly or indirectly. In this review, we provide a concise overview of the origin of mast cells and their distribution within cardiac tissue. We also discuss the critical role of activated mast cells in inflammation regression and tissue remodeling following cardiac injury. Furthermore, we highlight the dual roles of CMCs in cardiovascular disease development and their potential implications. Finally, we address future perspectives and directions in basic research, as well as the potential clinical applications of CMCs.

## Introduction

Cardiovascular diseases (CVDs) are the main cause of mortality and morbidity among non-communicable diseases worldwide. Although many factors have been associated with the occurrence of CVD, its pathogenesis is still unclear (Lauerer et al., 2024; Mao et al., 2025). As inflammation triggers the development of CVDs, the regulation of adaptive immune cell response through the production of inflammatory mediators such as interleukin 17A (IL-17A) (Wang et al., 2021), High-Mobility Group Box 1 (HMGB1) (Sun, Dong & Xiong, 2023; Lu et al., 2019), Angiotensin II (ANG II) (Lu et al., 2020; Lu et al., 2018), C-X-C motif chemokine ligand 4 (CXCL4) (Wei et al., 2024a; Wei et al., 2024b), which plays an important role in cardiac injury and tissue fibrosis, has been a focus of research. Therefore, in clinical or experimental data, the regulation of adaptive immune cell response has been extensively studied, which provided insights into the prevention and treatment of CVDs. However, while adaptive immunity regulation has been extensively studied, an increasing number of studies highlight critical roles of innate immunity-including mast cells-in CVD pathogenesis, which has been relatively underappreciated compared to other innate immune cells such as macrophages and neutrophils. For example, macrophages, as a functionally heterogeneous cell population, can undergo functional remodeling according to the microenvironment they are in, thus participating in tissue damage (Lu et al., 2018; Lin et al., 2024; Liu et al., 2024) and repair (Ruiz Luque et al., 2024). Neutrophils can release neutrophil extracellular traps (NETs) upon activation, which capture platelets and red blood cells, promoting thrombus formation and thereby exacerbating cardiovascular diseases (Geng, Wang & Li, 2024; Han et al., 2022). Dendritic cells also play a role in regulating inflammatory responses in cardiovascular diseases (Britsch et al., 2024). For example, in atherosclerosis, dendritic cells activate T cells by presenting antigens, which promotes the recruitment of inflammatory cells and the release of inflammatory factors, thereby exacerbating the instability of atherosclerotic plaques (Zhang et al., 2024c). In contrast, research on mast cells in cardiovascular diseases is relatively limited.

As non-circulating immune cells, mast cells (MCs) derived from bone marrow precursors mature under the influence of the c-kit ligand, stem cell factor (SCF), but their ultimate phenotype depends on the microenvironment in which they reside (Pahima & Dwyer, 2025; Deng et al., 2025; Poto et al., 2024). As underappreciated immune cells, they are generally considered as “allergic” effectors because of their pathophysiological roles in IgE-mediated skin (Macphee et al., 2024; Mehrani et al., 2024), airway gastric (Alvarado-Vazquez et al., 2024), and urinary tract allergic reactions (Atiakshin et al., 2024b). Activated MCs release histamine and proteases such as tryptase and chymase through degranulation and produce a variety of cytokines and growth factors, which are involved in the pathogenesis of various diseases and in Joulia et al. (2024), Karhausen et al. (2020), Huo et al. (2023) and Voelker & Pongdee (2025). Several studies have documented an association between increased cardiac mast cells (CMCs) numbers and cardiac pathological conditions, such as myocardial infarction (MI) (Ngkelo et al., 2016), hypertension (Atiakshin et al., 2024a; Ge et al., 2022), myocarditis (Varricchi, Marone & Kovanen, 2020), and pulmonary vascular remodeling (Moriyama et al., 2022) in human and experimental animal models, though the causal relationship requires further validation. Despite these observations, the precise roles of CMCs in heart diseases and their underlying pathogenesis remain to be fully elucidated. Therefore, in this review, we aim to characterize and elucidate the physiological functions and activation mechanism of CMCs. This work will provide a foundation for designing intervention strategies to maintain cardiac homeostasis and promote cardiac remodeling following cardiac injury.

## Survey/Search Methodology

We conducted a systematic search of the literature published between 2000 and 2024 using databases including PubMed, Web of Science, and Scopus. The search terms combined keywords related to cardiac mast cells (*e.g.*, “cardiac mast cells inflammation”) and cardiovascular diseases (*e.g.*, “mast cells myocardial infarction fibrosis”). Inclusion criteria were: (1) peer-reviewed articles, (2) studies focusing on the role of cardiac mast cells in cardiovascular injury, inflammation, or fibrosis, (3) published in English. Exclusion criteria included conference abstracts, non-research letters, and duplicate studies. The initial search yielded 312 records, of which 124 met the criteria after screening titles/abstracts and full-text review.

## Origin and Subclasses of MC Characteristics

MCs were first discovered by Friedrich von Recklinghausen in 1863 and identified in 1878 (Blank, Falcone & Nilsson, 2013). These cells originate from hematopoietic stem cells. When MCs first enter the peripheral circulation from the bone marrow, they are in an immature state and only mature upon migrating to specific tissues, such as vascular tissue or serous cavities (Gentek et al., 2018). Extensive research has been conducted on both murine models and humans to identify different populations of MCs. MCs differentiate into two major subclasses of mature tissue MCs: a constitutive subclass composed of connective tissue-type MCs, which are often located around venules and nerve endings, and a T cell-dependent subclass composed of mucosal MCs, which are found interepithelially in the gut and respiratory mucosa (Varricchi & Marone, 2020). Furthermore, recent evidence suggests that the expression of secretory granule proteases in both cell types is influenced by the local tissue environment in which the cells reside.

### Mediators of MCs

MCs are capable of producing large amounts of cytokines, growth factors, chemokines, and proteases. These mediators play a critical role in the pathogenesis of many diseases (Shu et al., 2025), such as cancer (Yin et al., 2025) and autoimmune diseases (Lei et al., 2024), as well as in tissue injury and remodeling (Liu et al., 2024). Histamine, chymase, and tryptase are major inflammatory mediators released by MCs, with histamine being the most extensively studied due to its diverse modes of action. Histamine is involved in a wide range of physiological and pathological processes, including immune responses (Liu et al., 2025), inflammation (Zhou et al., 2025), and even the inhibition of plaque formation (Mekke et al., 2021). Chymase, first discovered in MCs, functions as a potent angiotensin-converting enzyme (ACE) that catalyzes the conversion of ANGI to ANGII (Ferrario et al., 2021). This enzymatic activity is crucial in the renin-angiotensin system, which regulates blood pressure and fluid balance (Ferrario et al., 2023). Numerous preclinical studies have demonstrated that suppressing chymase release alleviates cardiac fibrosis following MI, suggesting a potential causal role of chymase in this pathological process (Jin et al., 2019). However, the specific mechanisms underlying chymase’s contributions to CVDs remain to be fully elucidated. Tryptase, a multifaceted natural protease, has garnered attention for its diverse biological activities. Recent studies have demonstrated that tryptase serves as a biomarker for predicting cardiovascular complexity in acute coronary syndrome (ACS). Moreover, tryptase levels in ACS patients are closely associated with the risk of major adverse cardiovascular events (MACE), particularly in those with higher cardiovascular complexity, where the risk is more pronounced (Xie et al., 2025). In research on abdominal aortic aneurysm (AAA), tryptase levels have been found to be significantly correlated with the annual expansion rate of AAA (Wang & Shi, 2012). High levels of tryptase significantly increase the risk of subsequent surgical repair and all-cause mortality in patients. Given these contrasting roles, it becomes evident that clarifying the functions of mediators involved in mast cell degranulation is crucial for devising effective cardiac remodeling strategies in the aftermath of cardiac injury. Consequently, these findings may pave the way for a novel therapeutic approach in the clinical management of CVDs.

The cardiovascular effects and clinical significance of the main MC-derived mediators are summarized in Table 1, which systematically clarifies the regulatory roles of different mediators in cardiac homeostasis and pathological processes.

**Table 1:** Mast cell-derived mediators: cardiovascular effects and clinical significance.

Mediator	Main Cardiovascular Effects	Clinical Significance/Research Evidence
Histamine	1. Regulates T cell immune responses in viral myocarditis 2. Participates in cardiac tissue inflammatory response 3. Inhibits atherosclerotic plaque formation	1. H1 receptor signaling is associated with susceptibility to coxsackievirus B3-induced myocarditis 2. Circulating histamine levels correlate with acute cardiac inflammatory injury severity
Chymase	1. ACE-independent generation of ANG II in humans and rodents 2. Stimulates TGF-β release to induce cardiac fibroblast activation and collagen synthesis 3. Promotes cardiac interstitial fibrosis after myocardial infarction (MI)	1. Chymase inhibition alleviates cardiac fibrosis and improves cardiac function post-MI 2. Cardiac chymase gene expression is upregulated in acute viral myocarditis and heart failure
Tryptase	1. Regulates TGF-β/SMAD pathway to promote cardiac fibrosis 2. Mediates abdominal aortic aneurysm (AAA) expansion	1. Serves as a biomarker for predicting cardiovascular complexity in acute coronary syndrome (ACS) 2. Tryptase levels correlate with AAA annual expansion rate and patient all-cause mortality 3. High tryptase levels increase the risk of major adverse cardiovascular events (MACE) in ACS patients
CXCL4	1. Regulates macrophage ferroptosis and inhibits CX3CR1+ macrophage phagocytic function 2. Aggravates cardiac dilatation and increases mortality in MI models 3. Promotes viral replication in viral myocarditis (VMC) by reducing type I IFN release 4. Activates TGF-β1/Smad2/3 signaling to induce cardiac fibrosis	1. CXCL4/NLRP3 axis is a key regulator of cardiac pyroptosis and fibrosis 2. MC-derived CXCL4 is a potential therapeutic target for VMC and post-MI cardiac remodeling
IL-6/IL-23	1. Promotes naive T cell differentiation into Th17 cells 2. Enhances Th17-mediated cardiac inflammatory injury in autoimmune myocarditis	1. MC-secreted IL-6/IL-23 is a critical link in adaptive immune-mediated cardiac injury 2. Targeting the IL-6/IL-23 axis may alleviate autoimmune myocarditis
TNF-α/IL-1β	1. Induces cardiomyocyte apoptosis and matrix metalloproteinase 9 (MMP9) deposition 2. Promotes cardiac fibrosis by enhancing inflammatory responses 3. Exacerbates myocardial tissue damage after cardiac injury	1. MC-derived TNF-α/IL-1β is a key mediator of early cardiac inflammatory injury 2. Inhibition of TNF-α/IL-1β signaling reduces cardiac fibrosis in rodent models

### Activators of MCs

**Figure 1:**
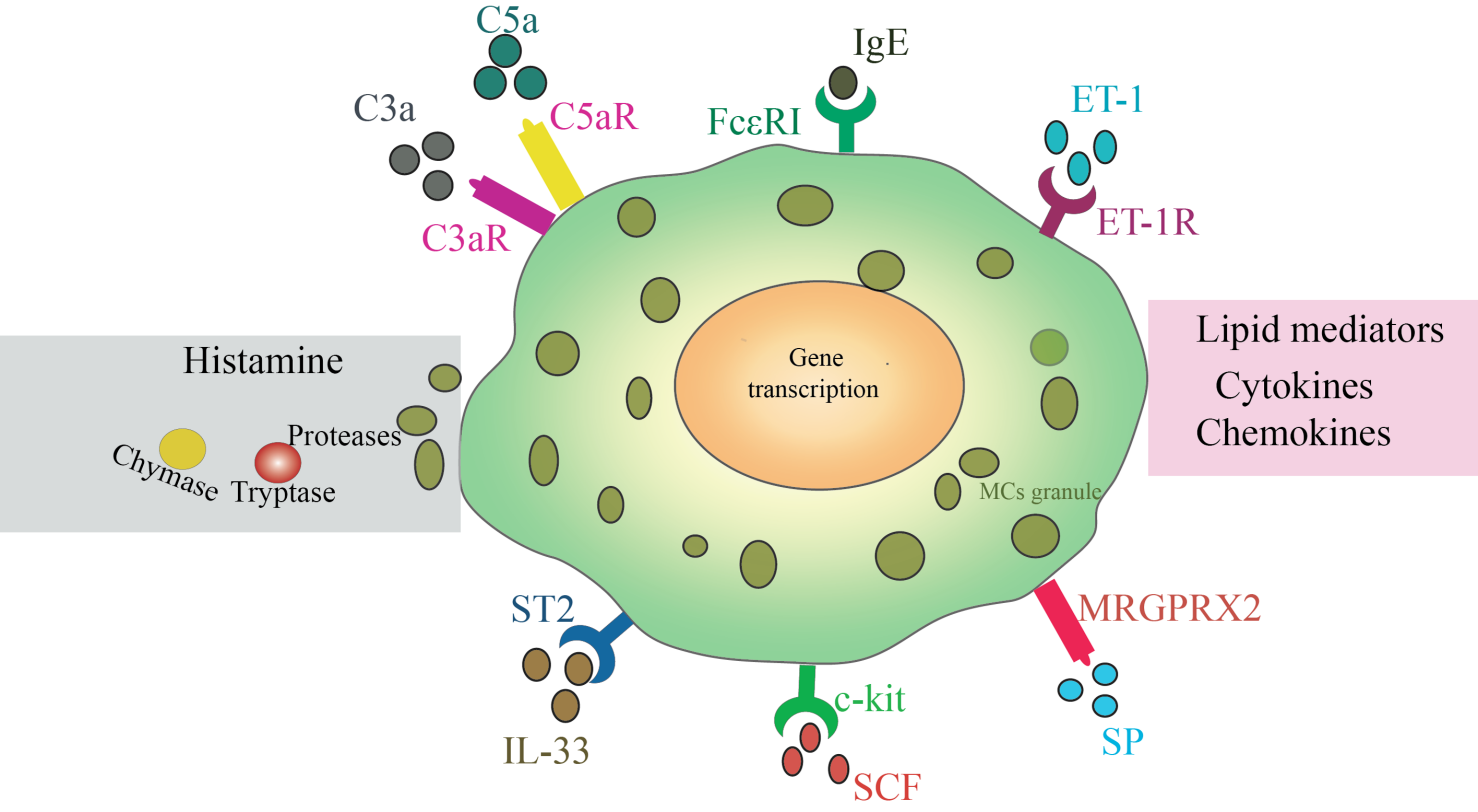
MCs can be activated by many kinds of stimuli. IgE mediated activation of Fc*ɛ* RI triggers exocytosis of granules, a rapid progressing and release of cytokines, chemokines and liquid mediators. IL-33, which activates the ST2 receptor also trigger release of cytokines, chemokines. ET-1 *via* ET-1R promotes MCs development. SP, as a powerful inflammatory mediator, It induces degranulation of MCs by binding to the MAS-related g-protein-coupled receptor member X2 (MRGPRX2) receptor expressed on MCs. C3a and C5a are by-products of complement activation, which are activated by binding to surface receptors of MCs. SCF *via* its receptor c-kit on MCs involved in the development of many diseases.

The best-known MC activation pathway is the binding of antigen-specific IgE to Fc*ɛ*RI. Besides Fc*ɛ*RI, MCs express other cell receptors for their activation, including toll-like receptors, complement 3a/5a receptors, and adenosine receptors (Fig. 1). Despite the critical role of CMCs in CVDs, the factors causing MC activation are poorly understood. Therefore, identifying the activators that target these receptors will provide insights into the mechanisms of CMCs in tissue injury and remodeling.

#### Complement 3a/5a

The complement system is crucial for innate immunity against bacteria and other pathogens. MC-mediated innate immunity may be closely related to the activation of MCs by complement molecules. C3a and C5a, by-products of complement activation, are released upon binding to the surface receptors on MCs, namely C3aR and C5aR. Furthermore, studies have shown that the absence of complement molecules can alleviate MI in mice, with C5a appearing to play a significant role in scar formation (Asare et al., 2024). Additionally, C5a can promote the release of histamine by MCs, thereby participating in tissue inflammation (Vahldieck et al., 2024). However, further research is needed to determine whether C5a activation in MCs is a key factor involved in cardiac remodeling.

#### SCF

SCF is a novel pluripotent cytokine that exerts its biological functions through its receptor, c-kit. SCF can regulate the growth and development of hematopoietic cells across various lineages and is involved in numerous pathophysiological processes (Zhou et al., 2017), either independently or in concert with other cytokines such as IL-1, IL-6, and granulocyte-macrophage colony-stimulating factor. Recently, the regulatory effects of SCF on MC survival, proliferation, and activation have garnered considerable attention. Moreover, SCF’s role in recruiting MCs to tissues and promoting the pathological process of tissue fibrosis has also attracted significant interest (Zhang et al., 2024b). Therefore, elucidating these pathological mechanisms may enhance our understanding of the role of CMCs in cardiac remodeling.

#### Endothelin-1

Endothelin-1 (ET-1), a potent vasoconstrictive polypeptide, is extensively expressed on the surfaces of both vascular endothelial cells and smooth muscle cells. It is a key player in the pathogenesis and progression of CVDs and diabetes mellitus (Ji et al., 2023). Notably, Data have reported that under cardiac pathological conditions, the expression of ET-1 receptors is significantly upregulated. They identified that intravenous degranulation of MCs is triggered, which subsequently leads to collagen deposition (Masi et al., 2025). Their findings demonstrated that blocking the biological function of ET-1 effectively reduces the number of MCs located in the left ventricle. This highlights the critical role of ET-1 in mediating cardiac fibrosis and suggests that targeting the ET-1 pathway may represent a possible strategy, though more studies are needed.

#### Substance P

Substance P (SP) is one of the earliest identified neuropeptides and is widely distributed throughout the body. It has been implicated in the pathogenesis of numerous diseases. Recently, SP has gained recognition as a potent inflammatory mediator. It induces MC degranulation by binding to the MAS-related G-protein-coupled receptor family member X2 (MRGPRX2) expressed on MCs (Nagamine et al., 2024). This binding triggers the secretion of histamine, chymase, and tryptase, which are involved in allergic reactions (Zhang et al., 2023), asthma (Li, Zhong & Xu, 2024), and tissue injury. A recent study has revealed that cardiac myocytes are sensitive to SP and play a significant role in CVDs (Chen et al., 2022). However, the specific underlying mechanisms remain to be elucidated.

#### IL-33

Interleukin-33 (IL-33), a member of the IL-1 cytokine family, plays a crucial role in both innate and adaptive immune responses by binding to its receptor ST2 (Zhou, Xu & Liu, 2023). ST2 is expressed on a variety of immune cells, including macrophages, neutrophils, basophils, MCs, and regulatory T cells (Sheng et al., 2025). IL-33-mediated immune signaling is involved in tissue inflammation and repair responses across various organs, such as the lungs, skin, and cardiovascular system. These finding providesnovel insights into the study of mast cell biology.

## Interaction of MCs with other immune cells

**Figure 2:**
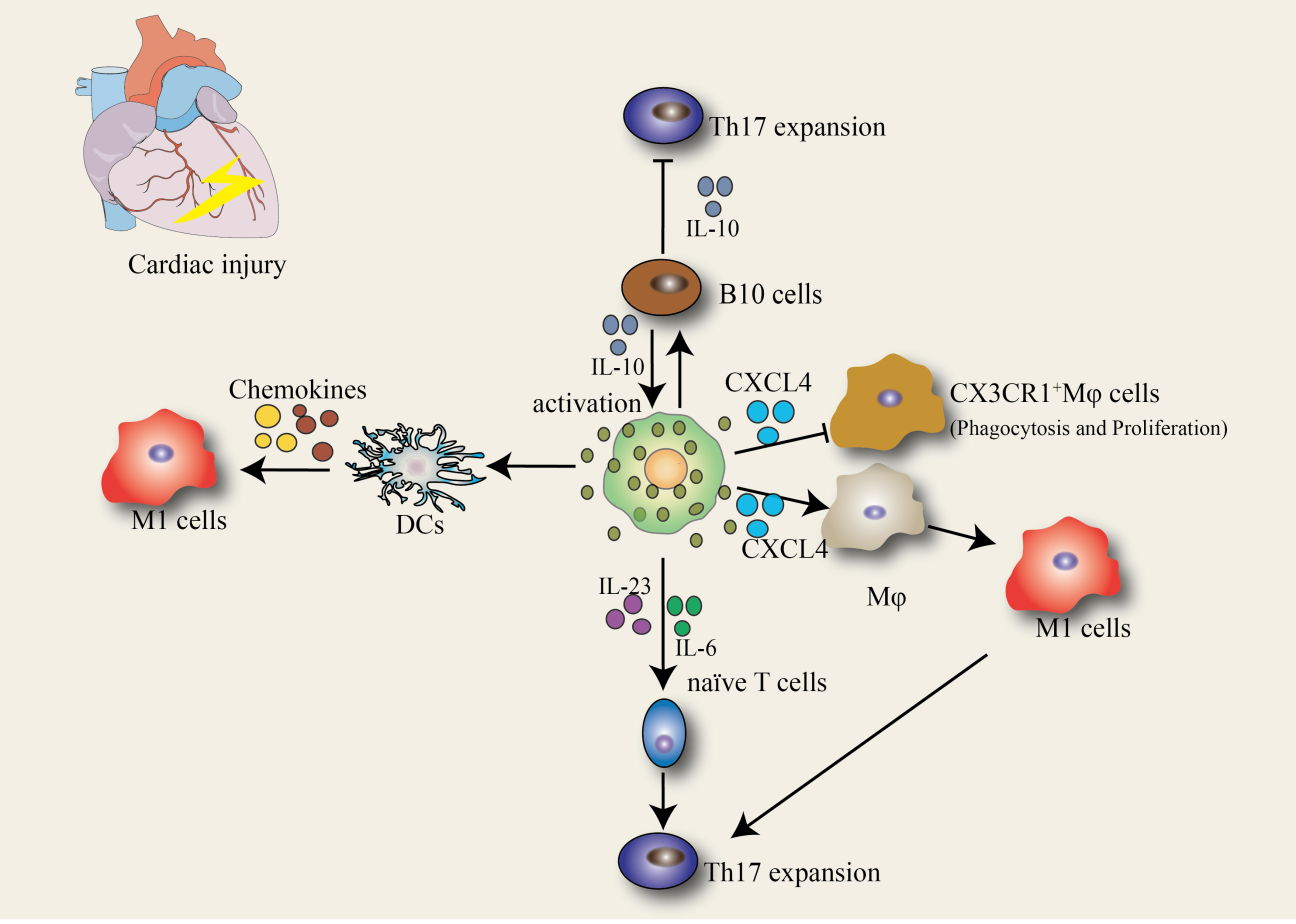
The crosstalk between MCs and various other immune cells. MCs engage in communication with multiple cell types, including T cells, B cells, macrophages, and dendritic cells (DCs). In the context of cardiac injury, as depicted in the schematic, a central MC hub orchestrates diverse, multifaceted interactions: with T cells, it secretes IL-23 and IL-6 to drive the differentiation of naïve T cells into Th17 cells, while also producing IL-10 to activate B10 cells that in turn suppress Th17 expansion, forming a regulatory loop; with B cells, MC-derived IL-10 activates B10 cells to exert immunoregulatory effects; with macrophages, it releases CXCL4 to promote the phagocytosis and proliferation of CX3CR1 + macrophages and induce the polarization of macrophages into pro-inflammatory M1 cells; and with DCs, signals from MCs prompt DCs to secrete chemokines that recruit M1 cells. These interactions not only contribute to pathogen surveillance, anti-pathogen immunity, and other mechanisms for eliminating microorganisms from the host but also play a pivotal role in shaping the immune landscape following cardiac injury, with examples of the functional consequences of mast cell communication and the mast cell mediators involved in target cell responses all being illustrated.

MCs can be selectively activated not only through the traditional receptor-mediated pathway but also by interacting with neighboring macrophages, T cells, B cells, and dendritic cells (DCs) and eosinophils. These interactions may influence the innate immune response to infection and trigger adaptive immune responses in other cells (Fig. 2).

### MCs and Macrophages

Macrophages play an important role in maintaining tissue homeostasis, tissue-specific function, and protecting tissues from stress. Moreover, the cells are involved in the pathogenesis of many diseases, such as cancers and autoimmune diseases, as well as in tissue damage and remodeling. Previous studies by our research group showed that a large number of inflammatory Ly6C^hi^ CCR2^+^ macrophages were involved in cardiac injury (Lu et al., 2018). During the differentiation of cardiac fibroblasts into myofibroblasts, these inflammatory macrophages were demonstrated to transform into Ly6C^low^ CX3CR1^+^ macrophages to participate in cardiac repair (Lu et al., 2020). Unfortunately, it is unknown whether the interactions between these three groups of cells contribute to the activation of CMCs, and whether activated MCs can inhibit these cells from participating in cardiac injury and remodeling. Our research data showed that MC degranulation causes the release of CXCL4 after cell activation. As a powerful pro-inflammatory mediator, CXCL4 promotes STAT3 phosphorylation and upregulates P53 expression to regulate macrophage ferroptosis, whereas it can inhibit the phagocytic function of CX3CR1^+^ macrophages, promote viral replication, and aggravate the pathogenesis of viral myocarditis (Wei et al., 2026). Therefore, elucidating the regulatory mechanisms will help researchers to propose novel treatment strategies for CVDs.

### MCs and T cells

MCs and T lymphocytes originate from bone marrow-derived pluripotent hematopoietic stem cells and play diverse roles in the immune system. In recent years, it has been shown that T cells play important regulatory roles in the generation, differentiation, and function of MCs. Moreover, the mediators released by MCs affect the function of T cells. Indeed, increased MC density and activation of MCs during T cell-mediated inflammation have been associated with delayed skin allergies (Chen, Griffiths & Bulfone-Paus, 2023), esophagitis (Htoo et al., 2024), Crohn’s disease (Sakita et al., 2022), viral infections (Hackler et al., 2023). During the pathogenesis of inflammatory disease, MCs secrete IL-6, IL-23, and other mediators directly or indirectly promote their differentiation into Th17 cells (Ruan et al., 2024).

### MCs and B cells

MCs have emerged as key regulators of adaptive immune responses, thanks to the growing understanding of the cross-reaction mechanisms between MCs and B lymphocytes. For many years, research on B cell/MC interactions has primarily focused on IgE production and allergic responses (Loste et al., 2023; Kim et al., 2015). Polukort et al. (2016) reported that IL-10 can directly promote the expansion, survival, and activation of MCs, thereby increasing Fc*ɛ* RI expression on MCs and enhancing the production of MC-derived cytokines. The discovery of B cell subsets with IL-10-dependent regulatory properties has broadened the scope of research into the mechanisms that control inflammation. Our group has found that B10 cells secrete IL-10 to inhibit Th17 differentiation, thereby alleviating cardiac injury (Chen et al., 2020). However, it remains unclear whether IL-10 secreted by B cells can promote the activation of CMCs and what role activated CMCs play during cardiac injury and repair.

### MCs and DCs

MCs and DCs are essential innate sentinel cells that surround host-environment interfaces. DCs are the most potent antigen-presenting cells and play a key regulatory role in innate and adaptive immunity. Current studies have shown that DCs can participate in the pathogenesis of autoimmunity and maintain homeostasis by controlling regulatory T cell expansion in many autoimmune diseases, such as experimental autoimmune encephalomyelitis (Zhuang et al., 2023), type 1 diabetes (Cobo-Vuilleumier et al., 2024), and systemic lupus erythematosus (Li et al., 2024). Leyva-Castillo et al. (2021) identified that the activation of MCs induces MHCII presentation on DCs during skin inflammation and that DCs secrete chemokines that recruit mononuclear macrophages to participate in cardiac injury (Forte et al., 2021). However, whether MCs can perform similar functions remains unclear. Therefore, it is important to investigate the roles of DCs and MCs in cardiac injury and repair.

### MCs and eosinophils

Eosinophils are key effector cells in allergic and inflammatory responses, and they form a bidirectional inflammatory partnership with mast cells in cardiac inflammatory microenvironments, with histamine serving as a central mediator of this crosstalk (Poto et al., 2024; Varricchi, Marone & Kovanen, 2020). On the one hand, activated mast cells release histamine *via* degranulation, which binds to H1 and H4 receptors on the surface of eosinophils to promote eosinophil chemotaxis, activation and survival in cardiac tissue; histamine further upregulates the expression of eosinophil granule proteins (*e.g.*, major basic protein, MBP) and pro-inflammatory cytokines (IL-5, IL-13), thereby amplifying cardiac inflammatory injury and promoting fibrotic remodeling in pathological conditions such as myocarditis and ischemic heart disease (Liu et al., 2025; Higuchi et al., 2008). On the other hand, activated eosinophils secrete a variety of mediators including IL-5, eotaxin (CCL11) and platelet-activating factor (PAF), which in turn enhance mast cell degranulation and histamine release, and upregulate the expression of Fc*ɛ* RI on mast cells to augment mast cell activation sensitivity (Htoo et al., 2024; Zhong et al., 2022). This positive feedback loop between mast cells and eosinophils mediated by histamine and other inflammatory factors exacerbates the persistent inflammatory response in cardiac tissue, and disrupts the balance of cardiac immune microenvironment. Notably, the interaction between mast cells and eosinophils in cardiovascular diseases is far less characterized than in allergic diseases, and the specific regulatory mechanisms of this bidirectional partnership in viral myocarditis, myocardial infarction and cardiac fibrosis remain to be further elucidated (Poto et al., 2024; Varricchi, Marone & Kovanen, 2020).

## CMCs and cardiac injury

Cardiac injury is a complex pathological process that is often accompanied by a large number of inflammatory cells, such as monocytes/macrophages (Liu et al., 2023; De Berge et al., 2025) T cells (Zhang et al., 2025), and MCs (Xiong et al., 2020; Rabkin & Tang, 2023). A heightened initial inflammatory phase can lead to adverse degradation and weakening of the cardiac wall and can potentially cause cardiac rupture. Our research group reported that during the early stages of cardiac injury, damaged myocardium releases danger signals, which facilitate the recruitment of Ly6C^hi^ CCR2^+^ monocytes to the injured sites to further differentiate into inflammatory macrophages, thereby promoting inflammation through the production of pro-inflammatory cytokines, and the recruitment of neutrophils further aggravates cardiac injury (Lu et al., 2020). Additionally, we found that high mobility group box protein 1 directly promotes Th17 cell expansion, whereas ANGII facilitates monocyte/macrophage polarization to M1 phenotype and induces a Th17-mediated immune response (Lu et al., 2018). The number of MCs, as a group of functionally heterogeneous cells, was significantly increased during cardiac injury; however, the underlying mechanism remains unclear.

### MCs as a promoter of cardiac injury

**Figure 3:**
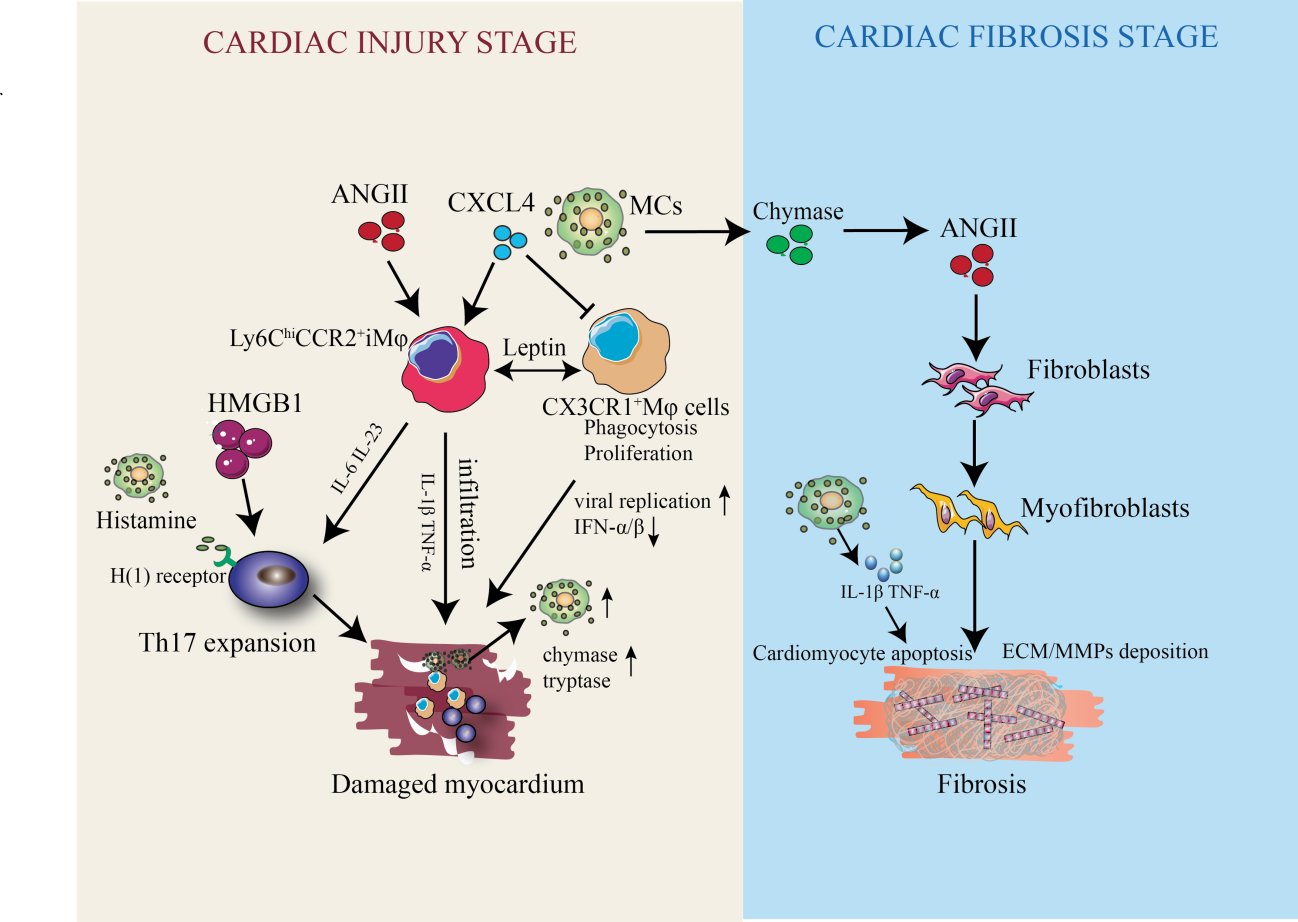
The dual-phase role of CMCs in cardiac injury and subsequent cardiac fibrosis. During the early cardiac injury stage, damaged myocardium releases danger signals (*e.g.*, HMGB1) to upregulate CMC populations, where CMC-derived CXCL4 regulates the polarization of Ly6C hi monocytes into pro-inflammatory M1-type macrophages and, along with cytokines like IL-6, IL-23 and histamine (acting *via* the H(1) receptor), promotes Th17 differentiation to drive cardiac inflammatory injury; in the viral myocarditis (VMC) mouse model, this CXCL4 also inhibits CX3CR1 + macrophage proliferation and phagocytosis, reduces type I interferon (IFN-α/β) release, and enhances viral replication to exacerbate VMC progression, while CMCs and other immune cells infiltrate the damaged myocardium, releasing IL-1β, TNF-α, chymase, and tryptase to amplify tissue damage. During the cardiac fibrosis stage, CMC-secreted chymase generates angiotensin II (AngII) to induce fibroblasts to differentiate into myofibroblasts for extracellular matrix (ECM) deposition, and CMCs also produce TNF-α and IL-1β during degranulation to promote cardiac fibrosis *via* cardiomyocyte apoptosis and the deposition of matrix metalloproteinases (MMPs) and ECM, collectively detailing the molecular players and signaling pathways through which CMCs mediate cardiac inflammatory injury and fibrotic remodeling.

MCs, like other inflammatory cells such as macrophages and T cells, are essential for mediating inflammatory responses (Tada et al., 2023). Upon activation, MCs rapidly release characteristic granules and hormonal mediators in the interstitium, including histamine, cytokines (IL-1, IL-6, IFN-*γ*, TNF-α) and MC-specific proteases (tryptase and chymase) (Table 1) (Kovanen & Bot, 2017; Hermans et al., 2019). Notably, the gene expression of chymase and tryptase is significantly upregulated in the acute phase of viral myocarditis and further increased in the sub-acute phase of heart failure (Higuchi et al., 2008; Molly, Elizabeth & Ellis, 2023). while histamine/H1 receptor signaling modulates T cell responses and is closely associated with susceptibility to coxsackievirus B3-induced myocarditis (Table 1). In addition, Our group found that MC-derived CXCL4 has been shown to regulate macrophage ferroptosis in our recent experimental study (Wei et al., 2026), supporting a mechanistic role in promoting cardiac inflammatory injury in viral myocarditis models. In a VMC mouse model, CXCL4 was found to inhibit the proliferation and phagocytosis of CX3CR1^+^ macrophages, thereby reducing the release of type I IFN and promoting viral replication for the progression of VMC (Fig. 3). In conclusion, the role of MC degranulation in cardiac injury should not be underestimated.

### CMCs and cardiac fibrosis

Cardiac fibrosis is characterized by excessive deposition of extracellular matrix proteins, which can distort myocardial structure and promote the progression of arrhythmias and cardiac dysfunction (Ghazal et al., 2025; Docshin, Panshin & Malashicheva, 2024). It affects the clinical course and outcome of patients with heart failure and is a key driver of heart failure. Several immune cells are involved in this process, including cardiomyocytes, fibroblasts, endothelial cells, lymphocytes, and MCs (Zaidi et al., 2021; Rao et al., 2025). CMCs are multifunctional immune cells that play a complex role in tissue homeostasis and disease, with their secretory products exerting both pro- and anti-fibrotic effects depending on microenvironmental signals.

### CMCs as a key regulator of fibrosis

Cardiac interstitial fibrosis is a characteristic sign of aging and is common in a variety of cardiac diseases. This fibrotic response further impairs cardiac function and promotes arrhythmia. In other fibrotic situations, myofibroblasts are the key cells that drive the fibrotic response. In a healthy heart, CMCs are located in the stroma near cardiac fibers and capillaries. Additionally, MC density significantly increases with degranulation during dilatation and ischemic cardiomyopathy (Legere et al., 2019). MC degranulation products have an important effect on fibrosis; however, the specific stimulation mechanism for degranulation remains unclear. The transforming growth factor β (TGF-β)/SMAD pathways play a vital role in inducing fibrosis (Pan et al., 2025; Zhang et al., 2024a). MC-derived chymase and tryptase trigger the release of the fibrogenic factor TGF-β to induce cardiac fibroblast activation, myofibroblast differentiation, and collagen synthesis. Chymase, as an ACE-independent ANGII generator, further promotes fibroblast-to-myofibroblast transformation and collagen deposition (Hada et al., 2022; Zhong et al., 2022; De Souza et al., 2025). Moreover, inhibition or reduction of ANGII by ACE resulted in decreased cardiac fibrosis. In addition to tryptase and chymase, MCs produce TNF-α and IL-1β during degranulation, which promotes cardiac fibrosis by inducing cardiomyocyte apoptosis, inflammation, and matrix metalloproteinase 9 deposition (Table 1, Fig. 3). However, a major limitation of current research is the lack of specific markers to distinguish pro-fibrotic *vs.* anti-fibrotic CMC subsets in human cardiac tissue: most studies rely on MC markers (c-Kit, Fc*ɛ* RI) and cannot isolate functionally distinct CMC populations (Varricchi, Marone & Kovanen, 2020; Docshin, Panshin & Malashicheva, 2024). Additionally, conflicting evidence exists regarding the timing of CMC-mediated fibrotic regulation: preclinical studies show that CMCs promote early pro-fibrotic remodeling after cardiac injury, but limited data support whether CMCs switch to an anti-fibrotic phenotype in the late reparative phase, and no longitudinal human studies have validated this dynamic functional shift (Poto et al., 2024; Varricchi, Marone & Kovanen, 2020). Degranulated MCs can be identified in most species by analyzing the expression of c-Kit, Fc*ɛ* RI, and MC-specific proteases, yet the clinical utility of these markers is limited—circulating tryptase/chymase levels do not consistently reflect cardiac MC degranulation or fibrosis severity in heart failure patients (Voelker & Pongdee, 2025; Xie et al., 2025).

## Conclusions and Future Directions

MCs are important cell subsets that maintain homeostasis and participate in innate immune response. Mast cells are important, but their role is complex, and therapeutic targeting is promising but not yet definitive. In recent years, it has been confirmed that MCs, as the first line of defense against external infection, settle in specific organs, but the cascade between MCs and tissues remains unclear. At present, no comprehensive studies exploring the role of MCs in cardiac injury exist, and most reports are limited to cardiac injury accompanied by the increase in MC population. This led the researchers to ignore the interaction between adaptive immunity and MCs. Notably, in MI models, administration of MC-derived CXCL4 aggravated cardiac dilatation and increased mortality rates. In addition, MC activation may release other effector molecules that stimulate adjacent immune cells to perform different biological functions in different cardiac microenvironments.

To advance the translational potential of cardiac mast cell (CMC) research, future work should focus on three specific areas: defining context-dependent CMC subsets (such as pro-fibrotic c-Kit^+^ Fc*ɛ* RI^+^ Tryptase^+^ Chymase^+^ subsets in myocardial infarction, CXCL4-secreting subsets in viral myocarditis, and mucosal *vs.* connective tissue-type subsets in hypertension) to clarify their disease-specific roles; developing targeted therapies for CMC-fibroblast/eosinophil crosstalk (such as chymase inhibitors blocking ANG II-mediated fibroblast activation, H1/H4 receptor antagonists disrupting histamine-driven eosinophil recruitment, or dual-targeting strategies for reciprocal activation in fibrosis); and translating MC-derived mediators into clinical biomarkers (standardizing tryptase assays for ACS prognostic stratification, validating CXCL4 as a viral myocarditis-specific marker, optimizing tissue-targeted chymase detection to address current limitations, and establishing histamine metabolite reference ranges for acute cardiac inflammation) with integration into multicenter studies and clinical risk assessment tools.

Notably, translating MC subset biology into clinical therapies requires leveraging emerging preclinical evidence to advance drug development and clinical trials: (1) Mast cell stabilizers (*e.g.*, cromolyn sodium, ketotifen)—traditionally used for allergic diseases—show promise in CVDs by inhibiting MC degranulation; future phase II/III trials should stratify patients by MC activation status (*e.g.*, elevated tryptase/chymase levels) to validate efficacy in post-MI fibrosis or viral myocarditis, as preclinical models demonstrate reduced cardiac inflammation with these agents (De Berge et al., 2025; Xiong et al., 2020). (2) Tryptase inhibitors (*e.g.*, nafamostat, APC366)—already in clinical development for asthma—hold potential for ACS and AAA; prospective trials could evaluate their ability to reduce MACE risk in ACS patients with high tryptase levels (Xie et al., 2025) or slow AAA expansion by targeting tryptase-mediated matrix degradation (Wang & Shi, 2012). (3) CXCL4 blockers (*e.g.*, neutralizing antibodies, small-molecule antagonists)—a novel class of therapeutics-warrant clinical validation in viral myocarditis, given our preclinical data showing CXCL4 inhibition reduces macrophage ferroptosis and viral replication (Wei et al., 2026); initial phase I trials should focus on safety and pharmacokinetics in patients with biopsy-proven viral myocarditis. (4) Subset-specific targeting strategies-the next frontier in MC-directed therapy-could involve developing agents against MC subset markers (*e.g.*, MRGPRX2 antagonists for pro-inflammatory subsets (Nagamine et al., 2024)) or combining subset-targeted drugs with conventional CVD therapies to maximize efficacy while minimizing off-target effects. These therapeutic approaches, rooted in MC subset biology and mediator signaling, address unmet clinical needs in CVDs-particularly for patients with refractory fibrosis or inflammation. Future clinical trials should prioritize patient stratification by MC-related biomarkers (*e.g.*, tryptase, CXCL4) to enhance trial success, bridging the gap between preclinical discoveries and clinical application.

Overall, clarifying these specific areas will help in developing effective strategies for the prevention and treatment of myocarditis and other cardiovascular diseases.

## Additional Information and Declarations

### Data Availability

The following information was supplied regarding data availability: This is a Literature Review.
